# Interleukin 6 promotes BMP9-induced osteoblastic differentiation through Stat3/mTORC1 in mouse embryonic fibroblasts

**DOI:** 10.18632/aging.204504

**Published:** 2023-02-01

**Authors:** Shi-Yu Wang, Jin-Hai Jiang, Si-Yuan Liu, Jie Zhang, Xiang Gao, Hang Liu, Kai-Xin Ke, Yue Jiang, Lu Liu, Bai-Cheng He

**Affiliations:** 1 Department of Pharmacology, School of Pharmacy, Chongqing Medical University, Chongqing 400016, China; 2 Key Laboratory of Biochemistry and Molecular Pharmacology of Chongqing, Chongqing Medical University, Chongqing 400016, China

**Keywords:** interleukin 6, bone morphogenetic protein 9, osteogenic differentiation, mammalian target of rapamycin complex 1, signal transducer and activator of transcription 3

## Abstract

Interleukin 6 (IL-6) plays a dual role in regulating bone metabolism, although the concrete mechanism is unclear. Bone morphogenetic protein 9 (BMP9) is one of the most potent osteogenic inducers, and a promising alternative for bone tissue engineering. The relationship between IL-6 and BMP9 in osteogenic differentiation remains to be elucidated, and the osteoblastic potential of BMP9 needs to be enhanced to overcome certain shortcomings of BMP9. In this study, we used real-time PCR, western blot, immunofluorescent stain, fetal limb culture and cranial defects repair model to explore the IL-6 role in BMP9-induced osteogenic differentiation in mouse embryonic fibroblasts (MEFs). We found that the rat serum level of IL-6 was increased in the dexamethasone-induced osteoporosis model, and IL-6 expression was detectable in several progenitor cells and MEFs. BMP9 upregulated IL-6 in MEFs, and the BMP9-induced osteoblastic markers were elevated by IL-6, but reduced by IL-6 knockdown. BMP9 and/or IL-6 both activated mTOR, and the IL-6 effect on BMP9-induced osteoblastic markers and bone formation were reduced greatly by mTOR inhibition. Raptor was up-regulated by IL-6 and/or BMP9 specifically, and the osteoblastic markers induced by IL-6 and/or BMP9 were reduced by Raptor knockdown. Meanwhile, Stat-3 was activated by IL-6 and/or BMP9, and the increase of Raptor or osteoblastic markers by IL-6 and/or BMP9 were reduced by Stat-3 inhibition. The Raptor promoter activity was regulated by p-Stat-3. Our finding suggested that IL-6 can promote the BMP9 osteoblastic potential, which may be mediated through activating Stat-3/mTORC1 pathway.

## INTRODUCTION

Bone morphogenetic proteins (BMPs) belong to transforming growth factor superfamily, and several BMP members exhibit osteogenic potential in mesenchymal stem cells (MSCs). Although BMP2 and BMP7 have excellent osteogenic ability, they also have certain serious shortcomings [1, 2]. BMP9 shows stronger osteogenic potential than BMP2 and BMP7 [3]. Thus, BMP9 may be a promising alternative candidate for bone regeneration medicine. Unfortunately, certain deficiencies also exist in the BMP9-induced bone formation, including the duration of bone maturation being too long and adipogenesis occurring simultaneously [4]. Therefore, the BMP9 osteogenic capability should be further enhanced to meet with the need of bone tissue engineering.

Myositis ossificans, occurred as a result of trauma or without evidence of trauma, is the most common heterotopic ossification (HO) form [5]. Although the concrete pathological mechanism is unclear, it is well known that inflammatory reactions play an important role in HO [6, 7]. Interleukin 6 (IL-6), a pro-inflammatory and anti-inflammatory factor, is involved in acute and chronic inflammation [8]. IL-6 exerts physiological function through binding with its receptor, the membrane receptor (mIL-6R) or the soluble receptor (sIL-6R) [9, 10]; and then activates three signals, including Janus kinase-signal transducer and transcription-3 (JAK-Stat3), Jak-Ras/Raf and mitogen activated protein kinases (MAPK), and phosphoinositol-3 kinase/Akt (PI3K/Akt) [8]. IL-6 may affect the bone metabolism by regulating the activity of osteoblasts, osteoclasts, osteocytes, and cartilage [11]. Thus, the IL-6 effect on bone metabolism is controversial [12, 13], which may due to its pro-inflammatory or anti-inflammatory effects, the context, or a different manner of signaling transduction [14–16]. In mesenchymal stem cells (MSCs), BMP9 activates PI3K/Akt/mTOR signaling, and its osteogenic capability can be reduced by this signal inhibition [17, 18]. However, the details about BMP9 and PI3K/Akt/mTOR remains unclear.

Cyclooxygenase-2 (COX-2) is a well-known pro-inflammatory factor. In fact, COX-2 was also involved in promoting bone fracture healing and bone formation [19]. Our previous study demonstrated that COX-2 was up-regulated by BMP9, and the BMP9 osteoblastic potential was inhibited by COX-2 knockdown [20]. HO was inhibited apparently by administrating non-steroidal anti-inflammatory drugs, such as COX-2 specific inhibitor, celecoxib [21]. As a pro-inflammatory factor, IL-6 can be up-regulated by COX-2 [22]. Thus, moderate inflammation may enhance the osteogenic differentiation in MSCs, which might be a new way to elevate BMP9 osteoblastic potential.

In the present study, we investigated the BMP9 effect on IL-6 expression, and the IL-6 role in BMP9-induced osteoblastic differentiation; meanwhile, we analyzed the possible mechanism of IL-6 for bone metabolism. This study may extend our knowledge about the IL-6 effect on bone metabolism, and provide a new strategy to promote BMP9 osteogenic potential, which may enhance bone tissue engineering development.

## MATERIALS AND METHODS

### Cell culture and chemical reagents

HEK293 was originally purchased from American Type Culture Collection (Manassas, VA, USA). Mouse embryonic fibroblasts (MEFs) were extracted from NIH mouse embryos as report [23]. Cells were cultured using Dulbecco’s modified Eagle’s medium (DMEM) containing 10% fetal bovine serum (FBS), penicillin (100 U/ml), streptomycin (100 μg/ml), and at 37° C in 5% CO_2_. Rapamycin (HY-10219) and Stat-3 specific inhibitor (HO-3837, HY-100453) were ordered from MedChemexpress (Monmouth Junction, NJ, USA). Primary antibodies against Runx-2 (sc-390351), OPN (sc-21742), p-Akt1/2/3 (sc-377556), Akt1/2/3 (sc-56878), p-mTOR (sc-517464), and mTOR (sc-293089) were ordered from Santa Cruz Biotechnology (Santa Cruz, CA, USA); primary antibodies against IL-6 (21865-1-AP), Rictor (27248-1-AP), and Raptor (20984-1-AP) were ordered from Proteintech (Wuhan, China); primary antibody against β-actin (AC026) was ordered from ABclonal technology (Wuhan, China).

### Construction of recombinant adenovirus

The recombinant adenoviruses used for this study, including IL-6, BMP9, siRNA oligos of IL-6 and Raptor, were constructed using AdEasy system [24]. To track viruses, the recombinant adenoviruses were tagged with green fluorescent protein (GFP) or red fluorescent protein (RFP). The products were designated as AdIL-6, AdBMP9, AdsiIL-6, and AdsiRaptor. The recombinant adenovirus expressing GFP only (AdGFP) was used as vehicle control.

### RNA extraction, transcription reaction, and real-time PCR

Total RNA was extracted with Trizol reagent (15596018, Invitrogen, Carlbad, CA, USA). The RNA quality and concentration were measured using Nanodrop One (Thermo Fisher Scientific, USA), and 1 μg RNA was used to prepare complimentary DNA using reversal transcription reaction (RT) following the protocol of RT kit (Cat. No. R037A, Takara). SYBR green Kit (B21202, Bimake, Shanghai, China) and Bio-Rad CFX Connect system were used for real-time PCR assay with the setting as follows: 5 min at 95° C, followed by 40 cycles at 95° C for 20 s, 60° C for 20 s, and 72° C for 20 s; melt curve 65° C to 95° C. The relative mRNA level was calculated using the 2^−ΔΔCt^ method. Data were normalized to expression of β-actin. Primers used for quantitative real-time PCR were shown in Table 1.

**Table 1:** PCR primers used in this study.

**Gene**	**Primer**	**Sequence (5′→3′)**
IL-6	Forward	AGACTGGGGATGTCTGTAGCT
	Reverse	GACAGGTCTGTTGGGAGTGG
OPN	Forward	TGCACCCAGATCCTATAGCC
	Reverse	CTCCATCGTCATCATCATCG
Raptor	Forward	GCCGCCGTCTCTATTCCC
	Reverse	CTTCCTCCCCGAGTCCCA
β-actin	Forward	TGCTGACAGGATGCAGAAGG
	Reverse	CGGACTCATCGTACTCCTGC
Raptor (ChIP)	Forward 1	TGGGACCTGTTAGCATTCTGTC
	Reverse 1	GGAGGGGAGTTAAGAGGGTAGT
	Forward 2	GATTTTGGTGCACTGCTCTGAG
	Reverse 2	CTGCAGATGTGTTCCTCAGAGT
	Forward 3	AGTGGGCATCAGGACTACAAAG
	Reverse 3	CTGCTAGCATCTGAGAGTCCAG

### Protein extraction and western blot assay

Medium was discarded and cells were washed twice with cold phosphate buffered saline (PBS, 4° C). Total protein was extracted with lysis buffer containing phosphatase inhibitors on ice. Protein concentration was measured with bicinchoninic acid (BCA) kit (P0010S, Beyotime, Changhai, China). Samples were denatured by boiling for 10 min, and then subjected to electrophoresis using sodium dodecyl sulfate polyacrylamide gel. Then, protein was transferred to polyvinylidene difluoride membrane, blocked using 4% bovine serum albumin (BSA) at room temperature for 2 h, and followed by regular western blot assay. Finally, proteins were developed using chemiluminescent kit (160072, Saimike Biotech, Chongqing, China), and data were obtained using Bio-Rad ChemiDoc XRS+ imaging system (Bio-Rad, Hercules, USA). Quantification was performed with ImageJ software.

### Alkaline phosphatase (ALP) activity assay

ALP activity was measured on day 5 and day 7 following the introduction of kit (P0321S, Beyotime, Shanghai, China). Plates were scanned and images were taken using microscope (IX53, Olympus, Japan). Quantification was performed with ImageJ software.

### Alizarin Red S stain

The matrix mineralization nodules were measured using Alizarin Red S stain. On days 14 and 21 after treatment, medium was discarded and cells were washed twice with PBS (pH 4.2). Then, cells were fixed using 4 percent paraformaldehyde at room temperature for 20 min and washed twice with PBS (pH 4.2). Finally, cells were stained with 0.4 percent Alizarin Red S as described previously [4]. Plates were scanned and images were taken using microscope (IX53, Olympus, Japan). Quantification was performed with ImageJ software.

### Immunofluorescent stain and confocal assay

Cells were seeded in confocal dish, and treated with AdGFP, AdBMP9, AdIL-6, or AdBMP9 combined with AdIL-6, respectively. After 24 h, cells were washed with PBS, fixed with 4% paraformaldehyde for 30 min and washed with PBS. Then, treated with 0.5% Triton X-100 for 20 min on ice, and washed twice with PBS. Next, blocked with 5% BSA for 30 min. Subsequently, cells were incubated with antibody against p-mTOR at 4° C overnight, washed twice with PBS and incubated with Dyligh649-conjugated secondary antibody for 1 h. Finally, cells were stained with 4, 6-diamidino-2-phenylindole for 10 min, washed with PBS twice, and sealed with anti-fluorescence quenched reagent (P0126, Beyotime, Shanghai, China). Images were taken using confocal laser scanning microscope (SP8, Leica, Germany).

### Fetal limb culture and cranial defects repair assay

This animal study was approved by the institutional animal care and use committee of Chongqing Medical University. For fetal limb culture assay, E18.5 embryos were dissected under aseptic conditions and the limbs were cultured in DMEM with 0.5% BSA. After 24 h, the limbs were treated with AdGFP, AdBMP9, AdIL-6, and AdBMP9 plus AdIL-6. On day 14, the limbs were fixed with 4% paraformaldehyde for 48 h, and decalcified with 10% ethylene diamine tetraacetic acid decalcification solution for 2 weeks.

For cranial defects repair assay, C57bl/6J mice (female, 4~6 weeks old, 16~18 g) were ordered from Animal Experimental Center of Chongqing Medical University (Chongqing, China). Mice were anesthetized with 2% pentobarbital sodium, the head was fixed, and a 3 mm hole was made on the left side of skull suture with an electric drill. The hole was filled with cells (pretreated with AdGFP, AdBMP9, AdIL-6, AdBMP9 combined with AdIL-6, or AdBMP9 combined with AdIL-6 and 100 nM rapamycin). Finally, the incision was sutured carefully. Four weeks later, all mice were sacrificed and the cranium specimens were collected for further analysis.

### Histological staining

Limbs and cranial defect repair specimens were isolated and fixed using 4% paraformaldehyde for 48 h, decalcified using ethylene diamine tetraacetic acid decalcification solution for 2 weeks, and embedded in paraffin. Sections were de-paraffined and rehydrated for H&E or Masson trichrome stain.

### Enzyme-linked immunosorbent assays (ELISAs)

Serum IL-6 level was detected with rat IL-6 ELISA kit (RX302856R, RUIXIN biotech). The assay was performed following the kit’s instruction. Absorbance was measured at 450 nm with a plater reader (Varioskan 3020, Thermo Fisher Scientific, USA).

### Statistical analysis

Results were repeated three times independently, and data were shown as means ± standard deviation. The statistical difference was calculated using two-tailed student’s t-tests or one-way analysis of variance, and the difference was considered as statistical significance if the P value is less than 0.05.

## RESULTS

### Effect of dexamethasone and BMP9 on IL-6 expression in MSCs

**Figure 1:**
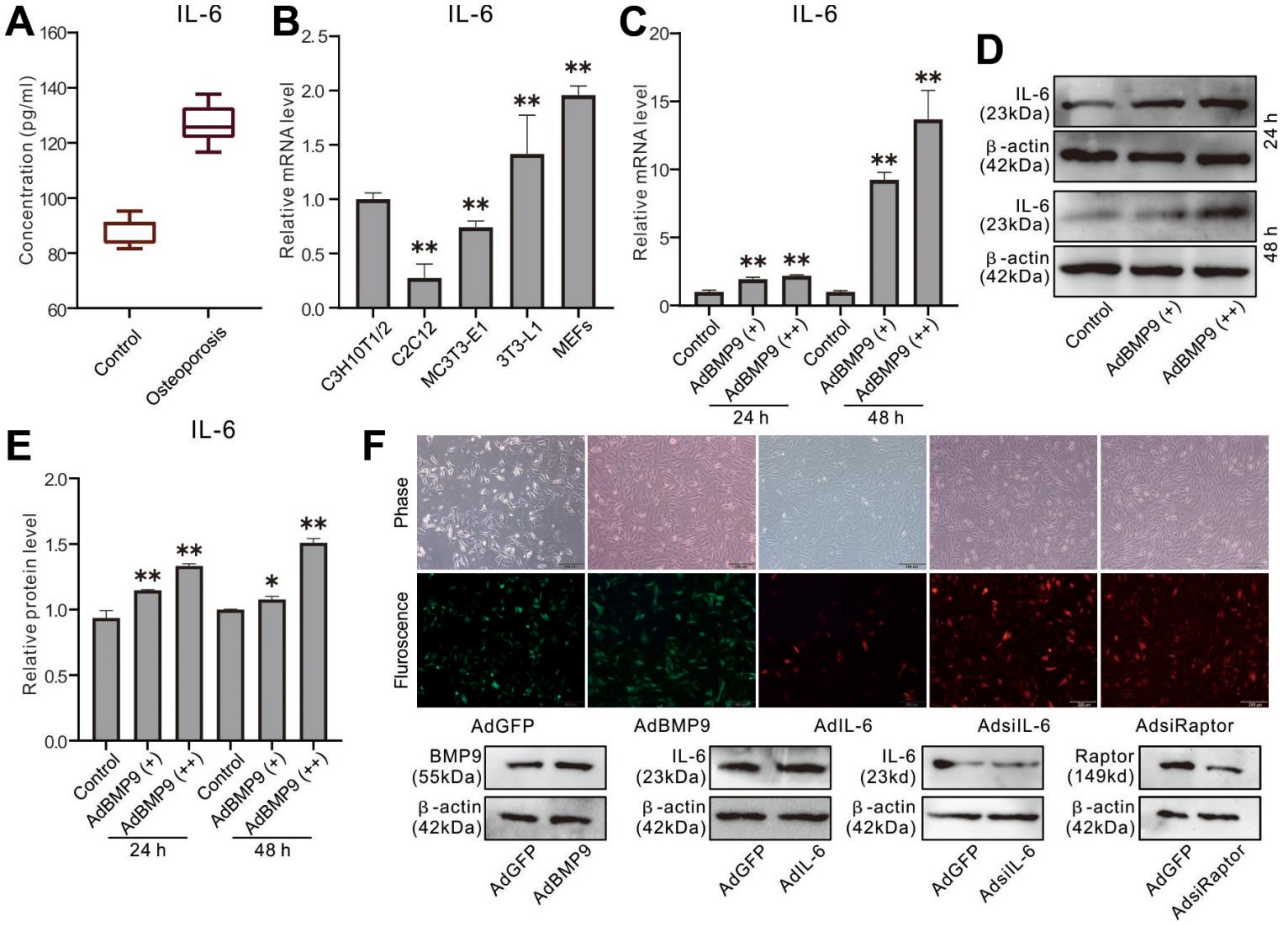
**Effect of osteoporosis and BMP9 on IL-6 Expression in MSCs.** (**A**) Rat serum level of IL-6 was measured with ELISA. (**B**) Endogenous IL-6 mRNA expression was measured with real-time PCR. (**C**) Effect of BMP9 on IL-6 mRNA expression was measured with real-time PCR. (**D**) Effect of BMP9 on IL-6 protein level was assayed with western blot. (**E**) quantification result of western blot assay shows the effect of BMP9 on IL-6 protein level. (**F**) The effect of recombinant adenoviruses on the target protein level were measured with western blot assay (upper panel show the infection of recombinant adenoviruses in MEFs). (^*^ *P* < 0.05, ^**^ *P* < 0.01).

We established rat osteoporosis model with dexamethasone, and the ELISA assay showed that the serum IL-6 level increased greatly in dexamethasone group than the control group (Figure 1A). Real-time PCR assay results showed that IL-6 mRNA is detectable in sever progenitor cells, and the level in MEFs is higher than the other cell lines (Figure 1B). Besides, BMP9 increased IL-6 mRNA and protein level in MEFs (Figure 1C–1E). These data suggested that IL-6 may be involved in bone metabolism and regulating BMP9-induced osteogenic differentiation, although the details remain unclear.

### Effect of IL-6 on the osteoblastic markers induced by BMP9 in MEFs

**Figure 2:**
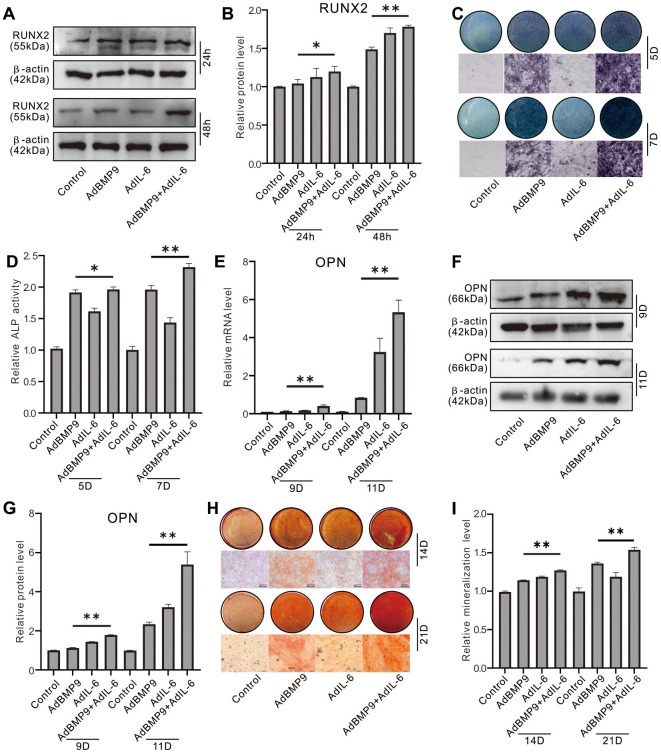
**Effect of IL-6 on the osteoblastic markers induced by BMP9 in MEFs.** (**A**) Effect of BMP9 and/or IL-6 on RUNX2 protein level were measured with western blot assay. (**B**) Western blot assay quantification shows the effect of BMP9 and/or IL-6 on RUNX2 protein level. (**C**) Effect of BMP9 and/or IL-6 on ALP activity was measure using histochemical stain. (**D**) Quantification of histochemical stain shows the effect of BMP9 and/or IL-6 on ALP activity. (**E**) Effect of BMP9 and/or IL-6 on OPN mRNA expression was measured using real-time PCR assay. (**F**) Western blot assay shows the effect of BMP9 and/or IL-6 on OPN protein level. (**G**) Quantification results of western blot assay show the effect of BMP9 and/or IL-6 on OPN protein level. (**H**) Effect of BMP9 and/or IL-6 on mineralization was measured using Alizarin Red S stain. (**I**) Quantification of Alizarin Red S stain show the effect of BMP9 and/or IL-6 on mineralization. (^*^ *P* < 0.05, ^**^ *P* < 0.01).

IL-6 enhanced the effect of BMP9 on inducing Runx2, although there was no obvious effect on Runx2 when IL-6 was used alone (Figure 2A, 2B). ALP activities were induced by BMP9 on day 5 and day 7, which were much stronger than those of IL-6; but the BMP9 effect on increasing ALP activity was enhanced substantially by IL-6 (Figure 2C, 2D). Similar results were found in OPN expression and matrix mineralization (Figure 2E–2I). These data suggested that IL-6 could promote the BMP9-induced osteoblastic differentiation synergistically in MEFs.

### Effect of IL-6 knockdown on the osteoblastic markers induced by BMP9 in MEFs

**Figure 3:**
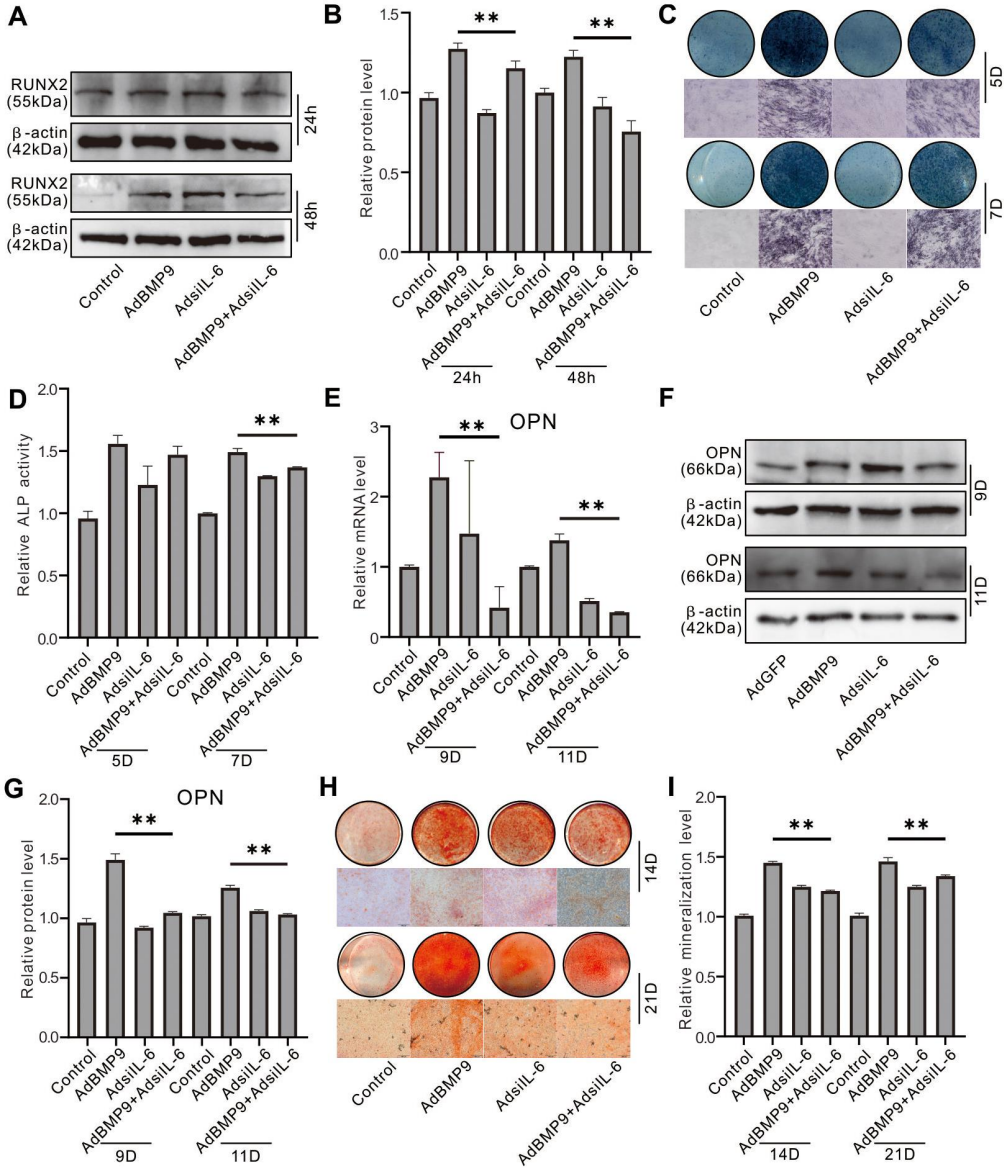
**Effect of IL-6 knockdown on the osteoblastic markers induced by BMP9 in MEFs.** (**A**) Effect of BMP9 and/or IL-6 knockdown on RUNX2 protein level were measured using western blot assay. (**B**) Western blot assay quantification shows the effect of BMP9 and/or IL-6 knockdown on RUNX2 protein level. (**C**) Effect of BMP9 and/or IL-6 knockdown on ALP activity was measure using histochemical stain. (**D**) Quantification of histochemical stain shows the effect of BMP9 and/or IL-6 knockdown on ALP activity. (**E**) Effect of BMP9 and/or IL-6 knockdown on OPN mRNA expression was measured using real-time PCR assay. (**F**) Western blot assay shows the effect of BMP9 and/or IL-6 knockdown on OPN protein level. (**G**) Quantification results of western blot assay show the effect of BMP9 and/or IL-6 knockdown on OPN protein level. (**H**) Effect of BMP9 and/or IL-6 knockdown on mineralization was measured using Alizarin Red S stain. (**I**) Quantification of Alizarin Red S stain show the effect of BMP9 and/or IL-6 knockdown on mineralization. (^**^ *P* < 0.01).

Western blot assay results showed that IL-6 knockdown reduced RUNX2 protein level which induced by BMP9 (Figure 3A, 3B). Histochemical stain results showed that the BMP9-induced ALP activities on day 5 and day 7 was inhibited significantly by IL-6 knockdown (Figure 3C, 3D). In addition, the BMP9-induced OPN and matrix mineralization were both decreased by IL-6 knockdown (Figure 3E–3I). These data indicated that the BMP9-induced osteoblastic differentiation may be reduced by reducing IL-6 expression in MEFs.

### Role of mTOR in mediating the IL-6 effect on promoting osteogenic differentiation in MEFs

**Figure 4:**
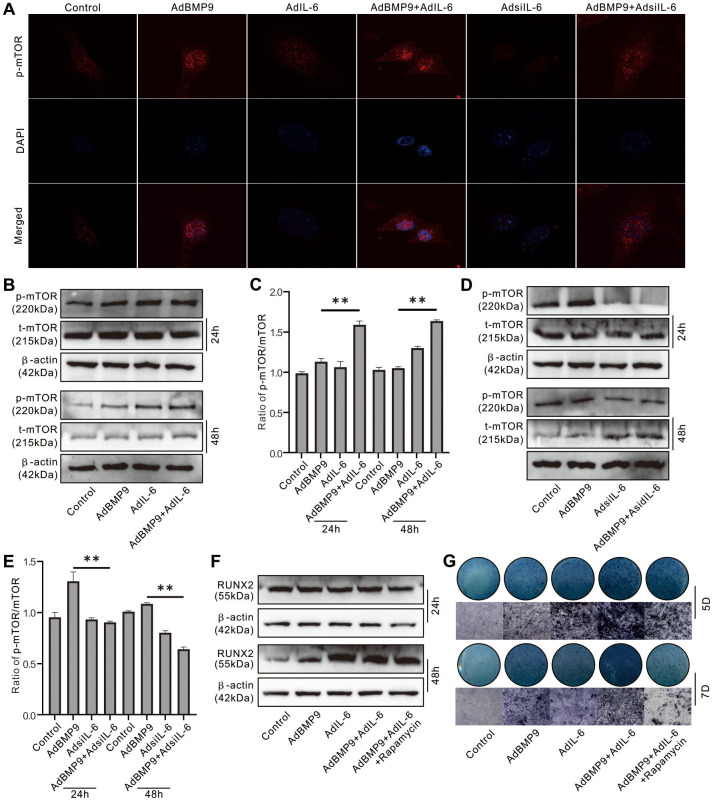
**Role of mTOR in the effect of IL-6 on promoting osteogenic differentiation in MEFs.** (**A**) Effect of BMP9, IL-6, and/or IL-6 knockdown on mTOR phosphorylation (p-mTOR) was measured using immunofluorescent stain and confocal assay. (**B**) Effect of BMP9 and/or IL-6 on total mTOR and p-mTOR level was measured using western blot assay. (**C**) Quantification of western blot assay show the effect of BMP9 and/or IL-6 on total mTOR and p-mTOR. (**D**) Effect of BMP9 and/or IL-6 knockdown on total mTOR and p-mTOR level was measured using western blot assay. (**E**) Quantification of western blot assay show the effect of BMP9 and/or IL-6 knockdown on total mTOR and p-mTOR. (**F**) Effect of IL-6 and/or rapamycin on BMP9-induced RUNX2 protein level was measured using western blot assay. (**G**) Effect of IL-6 and/or rapamycin on BMP9-induced ALP activity was measured using histochemical stain. (Rapamycin: mTOR inhibitor). (^**^ *P* < 0.01).

Immunofluorescent and confocal assay showed that BMP9 activated mTOR obviously, IL-6 exhibited moderate effect on activating mTOR but synergistically enhanced the mTOR activation induced by BMP9; IL-6 knockdown almost inhibited the mTOR activation, and the BMP9-induced mTOR activation was reduced by IL-6 knockdown apparently (Figure 4A). The level of phosphorylated mTOR (p-mTOR) was increased by BMP9 or IL-6, and the total mTOR level almost unchanged; but the p-mTOR level was significantly increased by the combination of BMP9 and IL-6 (Figure 4B, 4C). In contrast, IL-6 knockdown decreased the p-mTOR level, and the effect of BMP9 on increasing p-mTOR was inhibited by IL-6 knockdown notably (Figure 4D, 4E). In addition, the BMP9-induced Runx2 and ALP were increased by IL-6 over-expression, which were almost abolished by rapamycin (Figure 4F, 4G). These data suggested that IL-6 may promote BMP9 osteogenic potential through activating mTOR at least.

### Effect of IL-6 and/or rapamycin on BMP9-induced bone formation and bone defects repair

**Figure 5:**
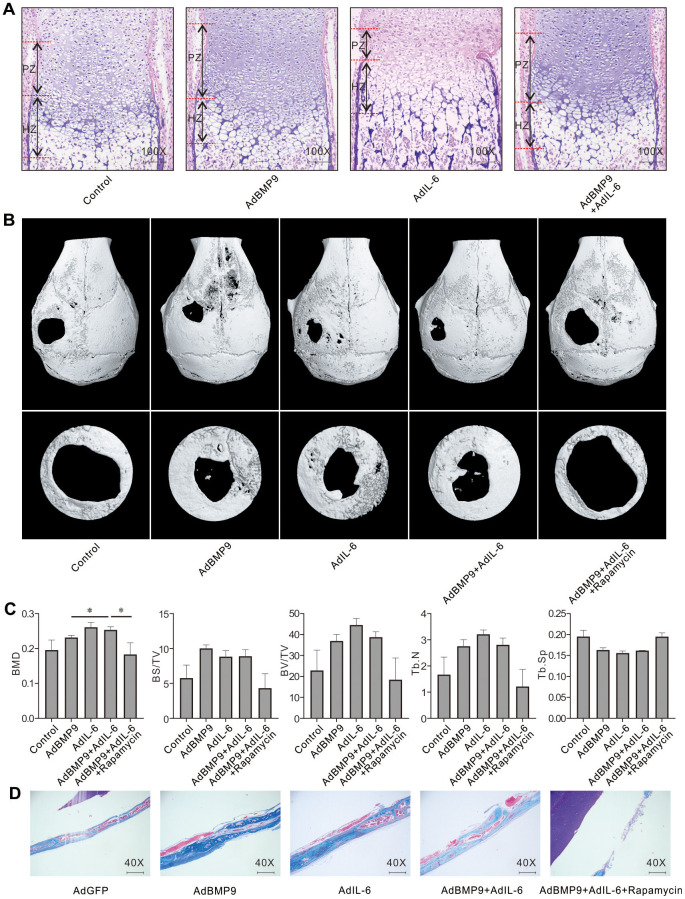
**Effect of IL-6 and/or rapamycin on BMP9-induced bone formation and bone defects repair.** (**A**) Effect of BMP9 and/or IL-6 on growth plate was measured using fetal limb culture (PZ: proliferation zone, HZ: hypertrophic zone; Scale bar: 250 μm). mTOR phosphorylation (p-mTOR) was measured using immunofluorescent stain and confocal assay. (**B**) Effect of BMP9, IL-6, and/or rapamycin on bone defects repair was measured using μ-CT scan. (**C**) Quantification analysis μ-CT scan shows the effect of BMP9, IL-6, and/or rapamycin on bone defects repair. (**D**) Effect of BMP9, IL-6, and/or rapamycin on bone defects repair was measured using Masson trichrome stain (Scale bar: 250 μm). (Rapamycin: mTOR inhibitor. BMD: Bone mineral density, BS/TV: Bone surface density, BV/TV: Percent bone volume, Tb.N: Trabecular number, Tb.Sp: Trabecular separation). (^*^ *P* < 0.05).

H&E stain showed that BMP9 increased the proliferation zone and hypertrophic zone compared to control group, and IL-6 increased hypertrophic zone but reduced proliferation zone; whereas the proliferation and hypertrophic zone were both increased by the combination of BMP9 and IL-6 (Figure 5A). Micro-CT analysis results showed that BMP9 or IL-6 both promoted the bone defect repair, and IL-6 exhibited no substantial effect on enhancing BMP9-induced repair; whereas, the effect of BMP9 and IL-6 on bone defect repair was greatly inhibited by rapamycin (Figure 5B, 5C). Masson trichrome staining results showed that BMP9, IL-6, or BMP9 plus IL-6 enhanced the bone formation, which was inhibited by rapamycin substantially (Figure 5D). These data also suggested that IL-6 and/or BMP9 may promote bone formation through activating mTOR at least.

### Role of Raptor in IL-6 effect on promoting osteogenic differentiation in MEFs

**Figure 6:**
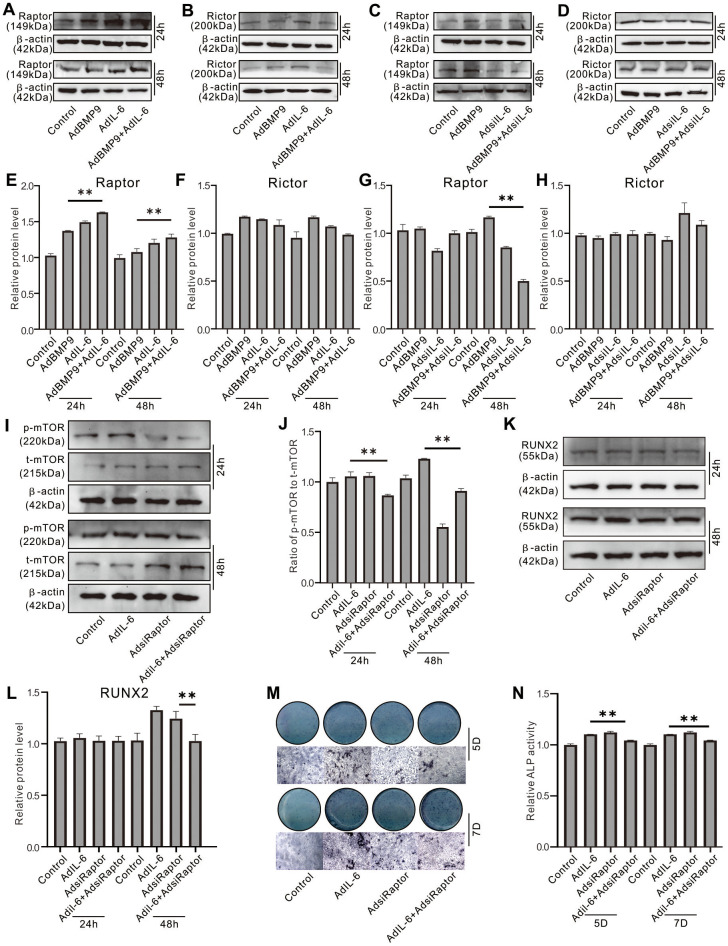
**Role of Raptor in the effect of IL-6 on promoting osteogenic differentiation inf MEFs.** (**A**) Effect of BMP9 and/or IL-6 on Raptor protein level was measured using western blot assay. (**B**) Effect of BMP9 and/or IL-6 on Rictor protein level was measured using western blot assay. (**C**) Effect of BMP9 and/or IL-6 knockdown on Raptor protein level was measured using western blot assay. (**D**) Effect of BMP9 and/or IL-6 knockdown on Rictor protein level was measured using western blot assay. (**E**) Quantification of western blot assay shows the effect of BMP9 and/or IL-6 on Raptor protein level. (**F**) Quantification of western blot assay shows the effect BMP9 and/or IL-6 on Rictor protein level. (**G**) Quantification of western blot assay shows the effect of BMP9 and/or IL-6 knockdown on Raptor protein level. (**H**) Quantification of western blot assay shows the effect of BMP9 and/or IL-6 knockdown on Rictor protein level. (**I**) Effect of IL-6 and/or Raptor knockdown on total mTOR and p-mTOR level was measured using western blot assay. (**J**) Quantification of western blot assay shows the effect of IL-6 and/or Raptor knockdown on total mTOR and p-mTOR level. (**K**) Effect of IL-6 and/or Raptor knockdown on RUNX2 protein level was measured using western blot assay. (**L**) Quantification of western blot assay shows the effect of IL-6 and/or Raptor knockdown on RUNX2 protein level. (**M**) Effect of IL-6 and/or Raptor knockdown on ALP activity was measured using histochemical stain. (**N**) Quantification of histochemical stain shows the effect of IL-6 and/or Raptor knockdown on ALP activity. (^**^ *P* < 0.01).

Western blot assay showed that IL-6 or BMP9 increased Raptor protein level, and IL-6 synergistically enhanced the BMP9 effect on Raptor (Figure 6A, 6E); whereas, both BMP9 and IL-6 exhibited no apparently effect on Rictor protein level (Figure 6B, 6F). In contrast, IL-6 knockdown decreased not only the level of Raptor, but also the BMP9 effect on increasing Raptor (Figure 6C, 6G); meanwhile, IL-6 knockdown showed no obvious effect on Rictor (Figure 6D, 6H). Raptor knockdown reduced the BMP9 effect on increasing p-mTOR level, but increased total mTOR level (Figure 6I, 6J). With the induction of osteogenic medium, IL-6 increased Runx2 protein level which was decreased by Raptor knockdown (Figure 6K, 6L). Similar results were also found in the matrix mineralization induced by IL-6 in MEFs (Figure 6M, 6N). These results suggested that IL-6 may promote the osteoblastic differentiation through activating mTORC1 via Raptor up-regulation in MEFs.

### Effect of Stat-3 on IL-6 induced Raptor in MEFs

**Figure 7:**
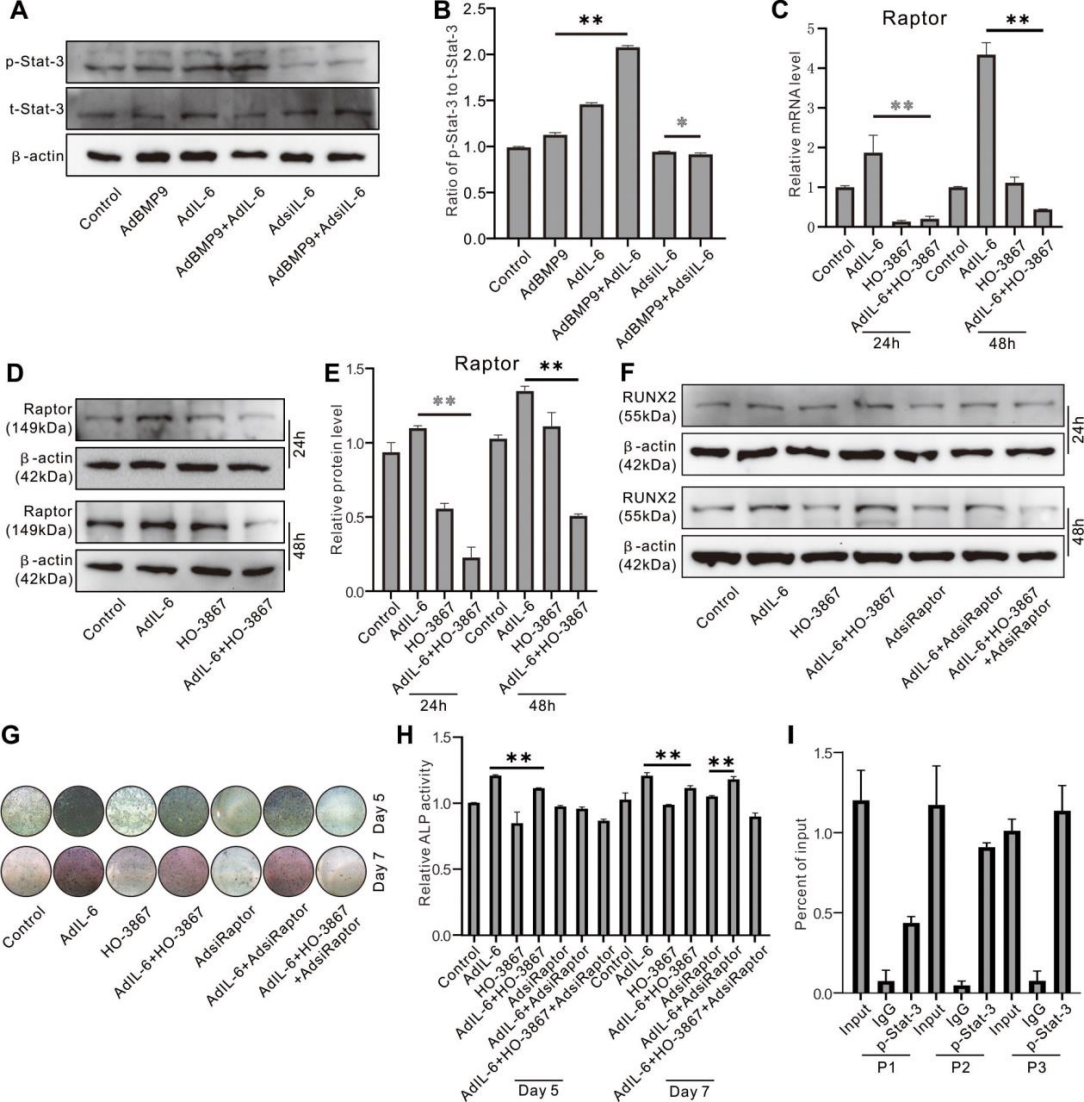
**Role of Raptor in the effect of IL-6 on promoting osteogenic differentiation inf MEFs.** (**A**) Effect of BMP9, IL-6, and/or IL-6 knockdown on p-Stat-3 and Sta3-3 was measured using western blot assay. (**B**) Quantification of western blot assay show the effect of BMP9, IL-6, and/or IL-6 knockdown on p-Stat-3 and Sta3-3. (**C**) Effect of IL-6 and/or Stat-3 inhibition on Raptor protein was measured using western blot. (**D**) Effect of IL-6 and/or Stat-3 inhibition on Raptor mRNA was measured using real-time PCR. (**E**) Quantification of western blot assay show the effect of IL-6 and/or Stat-3 inhibition on Raptor protein level. (**F**) Effect of IL-6, Stat-3 inhibition, and/or Raptor knockdown on Runx2 protein was measured using western blot. (**G**) Effect of IL-6, Stat-3 inhibition, and/or Raptor knockdown on ALP activity was measured using histochemical stain. (**H**) Quantification of histochemical stain show the effect of IL-6, Stat-3 inhibition, and/or Raptor knockdown on ALP activity. (**I**) Regulation of p-Stat-3 on Raptor promoter activity was measured using ChIP assay (P1: primer 1, P2: primer 2, P3: primer 3). (^*^ *P* < 0.05, ^**^ *P* < 0.01; HO-3867, Stat-3 specific inhibitor).

Western blot assay showed that IL-6 increased the level of phosphorylated Stat-3 (p-Stat-3), and BMP9 can also increase p-Stat-3 level; the p-Stat-3 level was synergistically elevated by the combination of BMP9 and IL-6 (Figure 7A, 7B). IL-6 markedly increased the Raptor mRNA level, which was reduced by Stat-3 inhibition (Figure 7C). Similar results were recaptured using western blot assay (Figure 7D, 7E). IL-6 increased Runx2 protein level, which was inhibited by Stat-3 inhibition or Raptor knockdown, respectively; the IL-6 effect on Runx2 protein was repressed more pronounced when combined Stat-3 inhibition and Raptor knockdown (Figure 7F). ALP assay showed the similar results (Figure 7G, 7H). ChIP assay results showed that Raptor promoter can be regulated by p-Stat-3 (Figure 7I).

## DISCUSSION

The IL-6 role in bone metabolism keeps controversial. In this study, we investigated the IL-6 effect on BMP9-triggered osteogenic differentiation in MEFs. We found that the BMP9-induced osteogenic markers and bone formation are promoted by IL-6, but inhibited obviously by IL-6 knockdown; the IL-6 effect on increasing BMP9 osteoblastic capability may be mediated by enhancing the activity Stat3/mTORC1 pathway partially at least.

Inflammation is implicated with bone metabolism [14, 25]. Dexamethasone and its derivates, termed as glucocorticoids, are a serial of excellent anti-inflammatory drugs [26]. Long term administration of glucocorticoids leads to osteoporosis, which known as glucocorticoid induced osteoporosis (GIOP) [27, 28]. GIOP may due to the increase of osteoclast activity, the decrease of osteoblast activity, or osteocyte apoptosis induced by glucocorticoids [29, 30]. Osteogenesis from stem cells may share similar physiological process with heterotopic ossification (HO) and vascular smooth muscle calcification. Inflammatory milieu is an important pathogenic cause of HO [6]; therefore, HO could be reduced by administration of nonsteroidal anti-inflammatory drugs, such as diclofenac and celecoxib [21]. Vascular smooth muscle calcification may also be induced by inflammation and can be reduced by meloxicam [31]. Thus, osteogenic differentiation may be promoted by moderate inflammation. However, it was reported that intestinal inflammation may lead to bone loss and fracture risk, which may duo to the high inflammatory cytokines level in bone marrow [32]; bone loss also occurred during periodontitis [33]. Therefore, inflammation may be a double-edged sword for bone metabolism, and the ultimate outcome may dependent on the inflammation severity and microenvironment.

Although BMP9 is the most potent osteogenic BMP, its osteogenic function still needs to be elevated. BMP9 may play a regulatory effect on inflammation by enhancing macrophage polarization through Nf-kB pathway [34]. The abnormal transition of endothelial-to-mesenchymal induced by BMP9 in endothelial cells was mediated by exacerbating inflammation via IL-6 [35]. If tumor necrosis factor-α (TNF-α) is available, BMP9 can recruit monocytes when combined with BMP10 [36]. BMP9 induced IL-6 and IL-8 expression in endothelial cells, and interaction of these factors with neutrophils and monocytes were also influenced by BMP9 [37]. Thus, BMP9 may be involved in promoting inflammatory reaction, and its osteogenic potential might be enhanced by enhancing inflammation moderately. Our previous study showed that BMP9 could activated PI3K/Akt/mTOR signaling, and its osteogenic potential was reduced by mTOR inhibition. PI3K/Akt/mTOR signaling activation is implicated with mediating TNF-α induced inflammation [38]. Therefore, the BMP9 effect on enhancing inflammation may due to PI3K/Akt/mTOR signaling activation. But the details underlying this process remain unclear.

IL-6, a well-known inflammation-related cytokine, exerts pro-inflammatory or anti-inflammatory effects through binding with membrane IL-6 receptor or soluble IL-6 receptor, then binding with glycoprotein 130 (gp130) [39]. The classic pathways activated by IL-6 include Janus protein-tyrosine kinases (JAKs), which in turn activates the signal transducer and activator of transcription (STATs, the predominant one is STAT3) pathway; other signaling cascades including mitogen-activated protein kinases (MAPKs), phosphoinositide 3 kinase/AKT (PI3K/AKT) and PKCδ [40–42]. Apart from regulating the immune function, IL-6 also regulates the activities of osteoblast, osteoclast and osteocyte [13]. Thus, IL-6 is one of the important pathogenic factors related to certain bone diseases, such as postmenopausal osteoporosis, and rheumatoid arthritis [13, 43, 44]. Since it may exert pro-inflammatory or anti-inflammatory effect, IL-6 exhibits dual effects on bone homeostasis [25, 45]. In this study, our data showed that IL-6 was up-regulated by BMP9 in MEFs, and the BMP9-induced osteogenic markers or bone formation were enhanced by IL-6 overexpression, but reduced by IL-6 knockdown. Hence, IL-6 may play an important role in regulating the BMP9 osteoblastic potential.

As the IL-6 mRNA and protein level were up-regulated by BMP9 in MEFs, we speculated that the BMP9 effect on PI3K/Akt/mTOR might be mediated by IL-6 partially. Our results showed that BMP9 or IL-6 both can activate mTOR, the BMP9-induced mTOR activation was enhanced by IL-6, but reduced apparently by IL-6 knockdown; the effect of BMP9, IL-6, or BMP9 plus IL-6 on increasing the osteogenic markers were inhibited by rapamycin obviously. The function of mTOR is mediated by two different complexes, namely mTOR complex 1 (mTORC1) and mTOR complex 2 (mTORC2); each complex comprises unique core component respectively. One unique component for mTORC1 is Raptor, and Rictor for mTORC2. In general, cell growth, protein synthesis and energy sensor are regulated by mTORC1, whereas mTORC2 mainly participates in regulating cytoskeleton, the activity of serine/threonine protein kinase or tyrosine protein kinase, such as Akt and insulin receptor [46–48]. Although the IL-6 effect on promoting BMP9 osteogenic potential was mediated through PI3K/Akt/mTOR signaling partially, it remains unknown which complex is associated with this function of IL-6. Further analysis showed that IL-6 and BMP9 increase Raptor protein level synergistically, but no substantial effect on Rictor; the mTOR activation induced by IL-6 was reduced by Raptor knockdown. IL-6 effect on inducing osteogenic makers was also reduced by Raptor knockdown. Thus, our data suggested that the IL-6 effect on promoting BMP9 osteogenic ability may be mediated by mTORC1 signaling via Raptor up-regulation. JAK/Stat-3 is another essential pathway through which IL-6 exerts its function. Our results showed that BMP9 and IL-6 both increased Stat-3 phosphorylation (p-Stat-3), and IL-6 enhanced the effect of BMP9 on increasing p-Stat-3 level, which suggested that the effect of BMP9 on increasing p-Stat-3 level may be mediated by IL-6. Further analysis showed that the IL-6 effect on Raptor was reduced by p-Stat-3 inhibition, and the IL-6 effect on promoting osteogenic differentiation was also reduced substantially by p-Stat-3 inhibition. Further ChIP analysis results showed the promoter of Raptor could be regulated by p-Stat-3.

**Figure 8:**
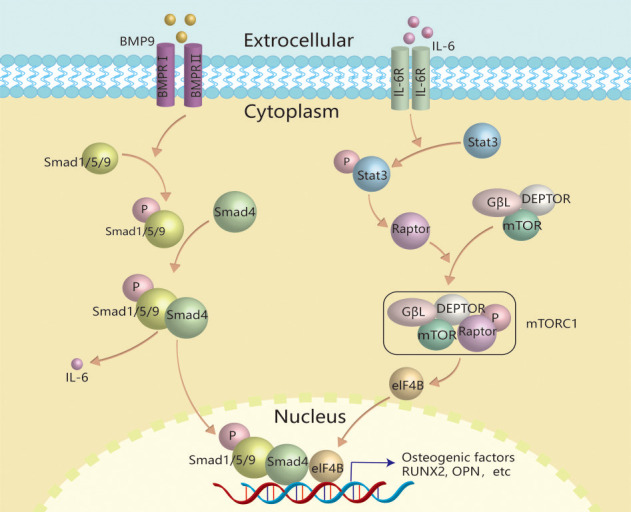
**Schematic diagram of IL-6 effects on promoting BMP9-induced osteogenic differentiation in MEFs.** IL-6 was up-regulated by BMP9 through binding with BMP type II receptor and type I receptor to activate BMP/Smad signal in MEFs. The secreted IL-6 binds with its receptor to activate the class pathway, and then promote the phosphorylation of Stat-3, through which to up-regulate the expression of Raptor. Then, Raptor binds with other components to form and activate TORC1, through which to activate the downstream transcriptional factor, such as eIF4B. Finally, the activated transcriptional factor may interact with the complex of p-Smad1/5/9 and Smad4 to promote osteogenic factor expression, such as RUNX2, etc.

Taken together, this study demonstrated that IL-6 may promote the BMP9 osteogenic potential through activating Stat-3/mTORC1 signal via up-regulating Raptor (Figure 8). Our findings suggested the IL-6 plays an important role in mediating the BMP9 osteogenic potential, and moderate inflammation stimulation may be a potent measure to accelerate bone formation.
